# The Philadelphia Bogus Diplomas

**Published:** 1881-04

**Authors:** 


### ARTICLE II.

### *The Philadelphia Bogus Diplomas.*

The confession of Buchanan, as published in the Philadelphia *Record*, announces the startling fact that no less than 60,000 bogus diplomas have been issued. It is gratifying to state that the diploma of the Baltimore College of Dental Surgery has never been sold or counterfeited. The confession is as follows:

"When Buchanan was entrapped, and the trick of his alleged drowning was completely exposed by his arrest in Michigan last September, the old man saw that his last hope of escape had vanished—so he accepted the inevitable and bowed to the supremacy of the law. On returning to this city he promised to do all that man could do to right the wrongs he had committed. He confessed judgment of ouster in the two suits brought by the Commonwealth to annul the charters of the Eclectic Medical College of Pennsylvania and the American University of Philadelphia. He also gave a power of attorney to confess judgment of ouster for him against the Livingstone University of America, chartered by the State of West Virginia, which charter has been annulled by the Legislature of West Virginia. He has given up all the books he had—the matriculation books, the minutes of faculty, minutes of trustees, account books, alumni minutes—and a mass of valuable information, including a list of foreign diplomas sold and a catalogue of addresses including over 5000 names of persons who had corresponded with him. He gives the names of wholesale druggists in Philadelphia who have sold his diplomas, and he gives the names of the parties to whom the diplomas were issued. He relates how diplomas were signed by the faculty; how in one instance three professors, for $5 each, signed 500 diplomas for him, and how, for $3.50, which were to go abroad were certified to by a Spanish Consul. In all, about 10,000 names were tangled up in his disclosures. He has given the names of many professional abortionists, and the means whereby they destroy life. He tells of the tricks of his trade, the quack nostrums that are advertised to cure all diseases, and of the impostors who prey on public credulity. He tells of faith in the supernatural; of a fortune-teller in Philadelphia who reads destiny under the light of candles made of human fat. He relates how one doctor goes to Europe annually and brings back love-powders which he represented were compounded at the shrine of Cupid in Minerva's temple. He describes how one concern sells the pulverized gizzard of a chicken as a compound to produce artificial digestion. He recites incidents wherein he robbed graves, and how on one Saturday morning he stole five dead bodies from Blockley Almshouse. He tells how he kept himself clear of the Courts and their penalties. He tells of twenty-five concerns in this country and in Europe by which degrees are sold. He figures that fully 20,000 bogus diplomas are current in America, and 40,000 more in Europe. He gives the authorities a lever by which they can uproot every diploma dealer in America.

As a result of *The Record's* expose of Buchanan's business the charters of the American University of Philadelphia, the Eclectic Medical College of Pennsylvania, the Philadelphia University of Medicine and Surgery, and the Livingstone University of America (at Charleston, W. Va.) have been annulled, and bills have been introduced into the State Legislature to repeal the charters of the Quaker City Business College, the Pen Medical University, the Philadelphia Electropathic Institution, and the Philadelphia College of Medicine.

Buchanan states: 'Before I became connected with the Eclectic College, I was respected. I was a member of Nazareth M. E. Church. I had a good practice, and was making a little money. After I joined the accursed thing I never made a dollar. During the last ten years my practice gradually left me—really I was making nothing. I had a heavy load to carry. Bread and butter had to be provided for three houses. The college was a mere farce—a sort of free school. I have struggled for ten years as no other man struggled. Once in I saw my error. Sites and Hollembach got me in, and long have I struggled to get out. I can assign no reason for my conduct. Insanity? Certainly, every act, when I look at it, has been that of a crazed fool. When told to run I ran. When two hundred diplomas were wanted I ordered them to be printed. I lost all self reliance. When the two hundred diplomas were burned they told me to go away, and I went away. If I had followed the dictates of my own mind I never should have done it.'

Buchanan's experiences with the law have been marked with many ups and downs. Between his Legislative investigation of 1872, his numerous suits at law, three arrests and escapes, his final capture and suppression by *The Record*, he spent large sums. The law disturbed him, but in a way it brought a compensating return. Speaking of the condition of the diploma market at a particular period, Buchanan says: 'There was no demand for diplomas in those days, as the Legislature was quiet. Stir up a Medical bill, then there is a demand.' He further says: 'The Legislative investigation of 1872 cost me $3000, which I paid to committeemen. One of my charters was repealed. The other one was not disturbed.

As soon as the repeal was announced, in the month of March, 1872, I sent one of my professors, Henry C. Stickney, to a lawyer named Tener. I gave Stickney all the points, and gave him Chillion B. Allen's diploma, as it was the only one we had which was fully signed. I knew that Allen had gone to Vienna, so I made up an account of tuition, board, loss of time, damages, etc., and sent all to Tener, with instructions to retain another lawyer—one to sue, the other to defend. It was to cost $300 for both—$100 down, and $200 if they got it through all right. I told them to keep away from Sharswood, as he was

University man. The result was a decision by Judge Agnew in our favor. Paine had closed up in consequence of the Legislative repeal, but this decision put new life in him. He imagined the same would suit both parties, so he opened again ; but I am sorry I did it.

In the abortion case of 1874 I paid $1250 to lawyers and $400 to my bail. The other cases were placed in the hands of a prominent lawyer, to whom I paid $250. Nothing more was heard of them until recently revived by Mr. Hagert.'

From Buchanan's confession and the books which accompany it is condensed what the Dean has to say about medical colleges :

*Eclectic Medical College of Pennsylvania* (*Philadelphia.*)—The minute books show that 'a meeting of the friends for the promotion of the science of medicine on the eclectic principle was held at No. 180 Vine Street on the 25th day of January, 1850.' This was the first entry made in the records of the institution. The sale of its diplomas dates back twenty-eight years, to 1853, and was continuously practiced from that date until the charter was annulled by the Courts six months ago. The faculty were cognizant of these doings. Their minutes show that they voted for and approved in many instances of the sale of their degrees. On the 15th of March, 1853, Dr. Simon Landis, of Ephrata, sent a letter announcing his intention of paying the full fees, $100, and submitting himself as a candidate for an illegal diploma. In 1863 appears the following :

The secretary having received a letter from—— ——, of England, wishing to know the price of a diploma, it was ordered that he be informed by the secretary that the diploma would be forwarded for $50 in English money. Professor Holtemback having also received letters from Dr. Rich, of Canada, relative to conferring degrees on several gentlemen in that province, it was ordered that Professor Hollembaek be authorized to communicate with the doctor and arrange the prices, etc.

Buchanan became acquainted with its faculty in 1856, and when Paine left, in 1860, Buchanan commenced to lecture. He says: 'I knew very little of the workings of the concern. The first sale of diplomas of which I was cognizant was that effected by Dr. Hollembaek in Canada.'

In 1868 Buchanan started the American University of Philadelphia, which was intended for the medical education of colored men. The Dean says: 'The entire faculty were radical copperhead Democrats, and for a few months I was compelled to leave the Pine Street college, owing to my degradation in lecturing to a negro class. I had seventeen dead bodies in the cellar that I had gathered together and owned. I stopped dissecting at the college, and that brought Sites and Hollembaek to terms. They sold out to me for $1000, $600 of which was paid to Sites and $400 to Hollembaek.'

To protect the college from Legislative interference Buchanan says he paid $50 to each of the thirteen members of the Educational Committee of the State Legislature in 1871 and $3000 to the Legislative investigating committee of 1872.

*The American University*, (*Philadelphia*,) was chartered in 1867. 'William Clark, of Cape May Court House, and myself got up the charter. One-half of the incorporators were his friends, chosen by him, one half was mine. It was a half arrangement all through. The college was designed for the education of the colored man, patronage and financial aid being promised by Congressman William D. Kelley, Bishop Simpson and others. I wrote out the charter, and called one Sabbath morning at the house of a State legislator, who I thought would be an available man for my purposes. He told me he would secure the charter for me, and I think I gave him some money that day; at any rate the consideration was to be $100. The charter went through rapidly. Clark paid $200, his half of the expense. I remember this well, because when I bought Clark out afterward, and paid him $100, he told me he lost $100. The copy of the charter I have given you is the one the legislator gave to me. It seems he charged $7.50 for it besides. We opened the colored school at Tenth and Chestnut Streets with thirty-six students. Clark, Hutchins, Parden, Terry, Roberts, Cochran and myself comprised the faculty. We carried through successfully two full lecture courses. Rev. J. T. Goodrich, D. D., of the church at Eighth and Noble Streets, was President, and had the books of this colored class with him when he was burned to death in the great Chicago fire. Up to this time we had received no pecuniary help from those who had promised to aid us.

From the year 1870 every student of the Eclectic College received an American University diploma as well as the Eclectic, and when the National Eclectic Medical Association was formed, in January, 1870, each student got a diploma of that society also. This was done to please the students. They wanted more sail.

Fully 15,000 diplomas of this university are scattered over Europe. One man—Dr. Rumler—now in New York, tells me that he sold 10,000 of our diplomas in Germany for Sturnam. I have given you a list of 1600 names of persons who were registered by us between 1871 and 1874. These were in addition to the 10,000.'

*Livingstone University.*—In regard to the Livingstone University of West Virginia, he says: 'There is a history attached to the stone on which the Livingstone diploma is now engraved. William Clark came to the college in 1863 as a lecturer on the Practice of Medicine. Having seen my diploma from Glasgow, he determined to have a copy of it, so he asked me to see a printer and have it copied, which was done. I think twenty-five copies were struck off. Clark got one, Cochran and Down also. I think Down paid a share of the engraving. Clark sold one to Dr. James Silverwright, of Chatham, Ontario. Then the stone was laid away in a cellar in a house in West Philadelphia. I subsequently bought the stone from Clark and had the Livingstone diploma engraved on it.

Professor Polk was in the office one day when Daniel Mayer, of West Virginia, arrived, saying he had got an order for 100 diplomas. Daniel was happy. Daniel Polk, Cochran, J. H. Brown and myself formed a circle of tables and signed all the diplomas.

At the college we sold some Livingstone diplomas for cash—not less than ten ; it may be over twenty ; several were given away to graduates, but none were sent to Europe by me. When Stickney left me in October, 1874, and went to Europe, I gave him fifty-three Livingstone diplomas ; but never heard from him until 1876, when he returned and apologized. As I was rich then, I left him in full possession of the college, which he afterward robbed.'

*Societies Controlled by Buchanan.*—In addition to the three charters which Buchanan owned there were quite a number of adjunct concerns in which his control was supreme. He owned the diploma plates of the Alumni Society of the Eclectic Medical College of Pennsylvania, of the National Eclectic Medical Association, and of the Pennsylvania State Eclectic Society. Buchanan organized in Western New York the Middle State Eclectic Society, in opposition to Robert S. Newton, of the Eclectic College of New York. He organized a society in opposition to the Brooklyn Academy of Medicine, also a department of dentistry to his college, and the University College of Pharmacy. Buchanan also furnished diplomas to an English Graduation Board, which had 400 names on its lists. Buchanan also issued diplomas upon the recommendation of the Maine Eclectic Society, and upon the recommendation of the President of the Eclectic Society of Boston.

*Philadelphia University of Medicine and Surgery.*—Originally the Philadelphia University was the American College of Medicine, which was chartered by the Legislature in 1853. In 1865 the name was changed to the Philadelphia University of Medicine and Surgery, and in 1872 the charter was repealed by the Legislature for diploma selling. Buchanan says that after the legislative investigation Paine's books were by mistake sent to the Pine Street concern, and Buchanan on examining them footed up a total of $90,000 for the sale of diplomas. Dr. Blood, who sells out patent rights of States for 'oxygenized air,' and who recently figured in the Christiancy divorce case, sold, according to his statement to Buchanan, one thousand of Paine's diplomas. Blood also sold for Buchanan.

*Penn Medical University.*—'Four lecturers in that concern,' says Buchanan, 'were connected in some way with our Pine Street college. One of its professors sold one of our diplomas to a Cuban for $200. Another of its professors, Alexander E. E. Falken, is on our list of graduates, although I believe he never took his diploma. W. H. Blake, one of the faculty, is also one of our men.'

*A Dental College.*—'When Dr. MacMichael was leaving this city to return to Canada he called on Asay, the toothmaker on Race Street, above Tenth, and bought quite a large order of teeth, which he intended to take to Canada. With the teeth he had a roll of six or ten diplomas, which he told me were obtained from a dental college in Philadelphia, and were to be sold to dentists in Canada. I saw the diplomas.'

'Edward James Gregory, a dentist of Cheltenham, England, assures me that he purchased two dental degrees for two friends in England for $100 apiece from the college at Tenth and Arch Streets.'

*Pittsburg and Bradford.*—'Dr. Sprecher, of Pittsburg, runs an Electrical Institute,' says Buchanan; 'I sold him three diplomas.'

'To Dr. Ginner, of the Bradford School of Medicine, I sent four or five certificates of membership in the National Eclectic Medical Association. Dr. Greiner applied to me for a foreign degree.'

*New York State Societies.*—'All physicians in New York must either register their diplomas with the Clerk of the County Court or they must pass an examination before one of the three State societies—Allopathic, Homœopathic and Eclectic. These are organized in every county. These societies grant certificates to any one who applies for authority to practice. They are terrible bleeders. I have known them to ask $300 to put a man through. New York City is exempt from all this—the Board of Health is the power there.'

*Eclectic Medical of New York City.*—'When Dr. Thomas Airy, of Manchester, England, was here in 1874, he bought a diploma from Robert S. Newton, of the Eclectic Medical College of New York, for a child of 17 years. I saw the diploma. I was asked to make this sale, but refused.'

'In 1871 Bowlsby, Newton's man, brought Rosenwig, a Jew, over to our college and got a diploma from Sites for him. Bowlsby stated that Newton had one ready for him. In those schools it is the first diploma that they look at with suspicion. Once graduated you can buy without affecting the scruples of the most fastidious. Then the diplomas are called *ad eundems*.'

'Among my express receipts you will find a record of a package sent to Dr. Kordenat, of Honesdale, Pa. That package contained a renewal of Kordenat's old diploma and one for his student, E. R. Hayden ; Kordenat had been promising to send this young man down to our college. Ultimately he said he could not do it, and told us plainly that if we did not like to sell the diplomas, Newton would.'

'I told Mr. Manwarren, of Mexico, New York, that I would take his son free at the college, but the father waited and waited, till at last he wrote to tell me that he had an excellent location for his son and he would like a diploma for the boy. He said he gave me the first show because I had offered him so liberally before. Newton had been to see him, he said, and would do it.'

'J. Phinnell bought a diploma from Dr. Sites, and having lost it he had it renewed. He told me he bought one from Fields, Newton and others.'

'Dr. S. P. Munn, of Waterbury, Connecticut, bought a diploma from us, although he never attended. He was President of the National Eclectic Medical Association, which was started by Newton and Wilder a year or more after William Young, of Newburyport, Massachusetts, inaugurated the original National Eclectic Association.'

'Alexander Wilder, Secretary of the National Eclectic Medical Association, says: 'degrees both genuine and spurious, have been notoriously a matter of traffic in this country for more than half a century, and I doubt now whether five colleges exist free from taint or imputation.'

*United States of New York.*—'This is a young concern, an offshoot from the Eclectics of New York. Before its organization the backers of this college offered to buy me out on Pine Street. There is one case in which I have personal knowledge of the sale of a diploma. They ask from $125 upward. I know that one of its officers offered to sell a diploma to N. W. Kinne, 32 West Jefferson Street, Syracuse, New York.'

*Druid University.*—'This concern is chartered by the State of New York, and extends all over the United States. Drs. Brown and Davies, of Athens, Maine, are owners of the charter, stone and seal. They adhere to Culpepper on Materia Medica. Davies was originally an Episcopal clergyman, but turned to medicine and took our diploma. The Druids are confined to the Welsh. The Druids implored me two years ago to abandon our State charter and begin under their auspices, but I was troubled with my head and would not go in. Last October a roll of their diplomas came to one of our students—Williams, of Maine. Somehow it got astray, and I traced it up for him. He told me what it was and whom they were intended for—parties who should not have such things. I asked him not to diliver till I wrote to Drs. Brown and Davies. It has been represented to me (I cannot vouch for it) that there is considerable money in this Druid affair. It is remarkable that the cactus grandiflorus, one of their remedies which operates on the heart, if given during the day causes suffocation and bleeding at the nose. If given when the sun sets it tranquilizes.'

*Trall's Water Cure College.*—' Trall's Water-cure College, at Florence, New Jersy, was chartered by New York State. I got quite a large number of students from Trall in 1868–'69–'70–'71, and we were quite friendly. Trall must have sold an unusual number of diplomas in England and America. I think Sturnam was his agent in England. In 1878 I received a letter from Sturnam, saying:

' I have also a number of Trall's diplomas and the right to grant them. I mean the Latin form—everything. Shall I send some? It has been hinted to me that Trall lately died. Now here's a chance for an old charter. Had you better not get it at once in another name or get some one to *secure* it? Write at once about this. I have his scholarship tickets—everything —and we could work the thing to pay us. •

The charter, too, of the old Metropolitan College of New York could be had. Write fully.'

*Western and Southern Institutions.*—'A doctor named Mumey or Hodgkins, bolonging to one of the St. Louis Medical Colleges, a refugee, whom I met last August at the sulphur springs of Windsor, Canada, told me that in the past five years 3000 diplomas of the college of which he was a professor had been sold in the West. He gave me the address of a party on Walnut Street, Kansas City, where he had made a sale. In his baggage he had eleven foreign diplomas in blank.'

*St. Louis Eclectic and American Eclectic.*—'With the exception of Fields, the Dean of the St. Louis Eclectic Medical, I think all the professors in the American Eclectic and St. Louis Eclectic are our graduates. While I have not seen any of their sold diplomas, yet I received from Sturnam in 1878 this offer:

'Some time ago you applied for 500 St. Louis diplomas. I can get you them. Their diploma is ridiculous—all in English—and I advised a new kind in Latin (not like yours). It is possible I shall have the making of the diploma. I have the appointment as the London branch.'

Thrasher, one of the faculty of the American Eclectic, sent me the following list of names, stating that the Physio-Medical College of Cincinnatti (Nicely's) had sold a diploma to each, and I could do likewise, as they were not pleased with the Cincinnati sheepskins: George T. Dougherty, F. M. Richardson, G. T. Ragan. G. W. Albur, J. W. McCartney, L. H. Mason, J. Richardson, Sr,. all of Neoga, Cumberland County, Illinois.

George T. Dougherty's name appears also in the list of graduates from the college with which Thrasher is connected, and the second name on the list, F. M. Richardson, is marked in their announcement as Dougherty's preceptor. It would seem from this that Dougherty has the diplomas of the American Eclectic of St. Louis, the American Eclectic of Cincinnati and the Eclectic of Pennsylvania, two of which were certainly bought. Thrasher, who is one of the rising men in the diploma business, got up a Michigan directory of doctors. I gave him a diploma in consideration of an advertisement he gave us. When he was at Nicely's I sent him another for a student.

'Rev. George Sexton, of London, who was prominent in the British Medical Reform Association, to whom I sold diplomas in 1874, is a European agent of Fields'. Sexton commenced his career as a missionary, then became a lecturer for Kahn, and now is the agent for the Anthropological University of St. Louis.'

*Anthropological University of St. Louis.*—Fields, Sexton, Alford and Thrasher have an organization as perfect as the Catholic Church. They have penetrated every city of America and Great Britan with their agencies. The Eclectic is not popular and does not sell well, but the University of Anthropology goes off like hot cakes. I have understood I could buy 500 of its diplomas in bulk at any time. They deal largely. Alford and Sturnam have approached me, but I never answered their communications.

The Scotch ministers take to these university degrees like a set of hungry wolves. They will not investigate. They have the degree mania badly. Fully 20,000 diplomas of this University have been sold in Europe to ministers, doctors and teachers.

Buchanan was Secretary of the concern at its organization. Alford was a lecturer at Buchanan's college, and the American University then embraced the Anthropological University as its theological branch.

*Cincinnati.*—Buchanan says he bought a diploma last year from W. R. Nicely, Dean of the American Eclectic College of

Cincinnati, Ohio, and Dean of the "Physio-Eclectic" of the same city.

*Boston.*—Concerning the New England University he says: 'Henry C. Stickney, who runs the concern, told me he had sold thousands of these diplomas throughout Europe. They were worthless.'

*Eclectic of Georgia.*—The Eclectic Medical of Georgia was the only Eclectic College in the country which was recognized by Buchanan as in communion with his National Medical Association. According to the minutes I. J. M. Goss, of this college, got a diploma from the Pine Street college, on the 25th of May, 1857 for $25. Fishblatt, who is credited as a Professor of Dermatology in that institution, was, Buchanan says, in an arrangement with Professor Polk to sell Eclectic diplomas to W. B. Dyer and twelve others. Dyer's diploma was returned, Dyer paying the express charges. The price was to have been $50.

*Keokuk, Iowa.*—'I was offered the chair of surgery in the Eclectic College at Keokuk, Iowa' says Buchanan, 'and they frankly told me how they did business. They make no secret of it. They sell like a grocer's shop. There are several thousand of their diplomas out in the West!'

*Detroit.*—Dr. Buchanan says two-thirds of the physicians of Detroit hold bought diplomas. The Michigan Eclectic College is a latent concern, in which Buchanan holds stock. At its head is H. S. Thomas, who cavorted around Detroit with the old man after the alleged drowning occurred, and swore to false affidavits regarding Buchanan's whereabouts at the time of the suicide ruse. Chapman says that Thomas was prepared to prevent Buchanan's return to Philadelphia if the Dean had been captured in Detroit, and that the programme was to swear that this man was not Dr. Buchanan, but Thomas' father.

*California.*—'I sold a diploma in 1871 to J. H. Bundy, Professor of a California Medical College. He paid $60.'"

